# Imaging Features of Alveolar Soft Part Sarcoma: Single Institution Experience and Literature Review

**DOI:** 10.3390/clinpract13060123

**Published:** 2023-11-01

**Authors:** Paolo Spinnato, Nicolas Papalexis, Marco Colangeli, Marco Miceli, Amandine Crombé, Anna Parmeggiani, Emanuela Palmerini, Alberto Righi, Giuseppe Bianchi

**Affiliations:** 1 Diagnostic and Interventional Radiology, IRCCS Istituto Ortopedico Rizzoli, 40136 Bologna, Italy; 2 Department of Orthopaedic Oncology, IRCCS Istituto Ortopedico Rizzoli, 40136 Bologna, Italy; 3 Department of Musculoskeletal Imaging, Pellegrin University Hospital, University of Bordeaux, 33000 Bordeaux, France; 4 Osteoncology, Bone and Soft Tissue Sarcomas and Innovative Therapies Unit, IRCCS Istituto Ortopedico Rizzoli, 40136 Bologna, Italy; 5 Department of Pathology, IRCCS Istituto Ortopedico Rizzoli, 40136 Bologna, Italy; 6 Orthopaedic Oncology Unit, IRCCS Istituto Ortopedico Rizzoli, 40136 Bologna, Italy

**Keywords:** magnetic resonance imaging, ultrasonography, multidetector computed tomography, soft tissue sarcomas, diagnosis, prognosis, sarcoma, alveolar soft part

## Abstract

Alveolar soft part sarcoma (ASPS) is an extremely rare and aggressive soft-tissue sarcoma (STS) subtype with poor prognosis and limited response to radiation therapy and chemotherapy. Prompt recognition and referral to sarcoma centers for appropriate management are crucial for patients’ survival. The purpose of this study was to report ASPS pre-treatment imaging features and to examine the existing literature on this topic. Twelve patients (7 women, 5 men—mean age 27.1 ± 10.7 years) were included from our single-center experience. Ultrasonography (US), computed tomography (CT), and magnetic resonance imaging (MRI) available were reviewed according to an analysis grid incorporating features from the latest research on STS. Clinical, histological, and outcome data were collected. MRI was available in 10 patients (83.3%), US in 7 patients (58.3%), and CT in 3 patients (25%). Mean longest tumor diameter was 7.6 ± 2.9 cm, and all tumors were deeply seated. Large peritumoral feeding vessels were systematically found and identified on ultrasonography (7/7), MRI (10/10), and CT (3/3). US revealed a well-defined heterogeneous hypoechoic pattern, with abundant flow signals in all patients (7/7). In all patients, MRI showed mildly high signal intensity (SI) on T1-WI and high SI on T2-WI and peritumoral edema. Moreover, flow-voids (due to arteriosus high-flow) into the peritumoral/intratumoral feeding vessels were detected in the MRI fluid-sensitive sequences of all patients. At baseline, whole-body contrast-enhanced CT revealed metastases in 8/12 (66.7%) patients. A pre-treatment longest diameter > 5 cm was significantly associated with distant metastases at diagnosis (*p* = 0.01). A maximum diameter > 5 cm represents a risk of metastatic disease at diagnosis (odds ratio = 45.0000 (95% CI: 1.4908—1358.3585), *p* = 0.0285). In the comprehensive literature review, we found 14 articles (case series or original research) focusing on ASPS imaging, with a total of 151 patients included. Merging our experience with the data from the existing literature, we conclude that the hallmark of ASPS imaging at presentation are the following characteristics: deep location, a slight hyperintense MRI SI on T1-WI and a hyperintense SI on T2-WI, numerous MRI flow voids, high internal vascularization, and large peritumoral feeding vessels.

## 1. Introduction

### 1.1. General Characteristics and Epidemiology 

Alveolar soft part sarcoma (ASPS) is an extremely rare soft-tissue sarcoma (STS) subtype, first described by Christopherson et al. (1952) [1]. It accounts for less than 1% of all STSs, usually affecting the extremities of children and young adults [2]. It is a very aggressive tumor characterized by poor prognosis, as well as a scarce response to radiation therapy and cytotoxic chemotherapy [2,3,4]. Surgical resection is the standard treatment based on complete resection with wide margins. Median survival time is about 3 years in metastatic patients at baseline versus 11 years in patients with locally advanced tumors only [2,3,5]. 

As for other STSs, symptoms are nonspecific and variable, depending on the affected sites and anatomical structures involved. A painless swelling or lump is one of the most common clinical presentations. Pain or soreness may be present in some cases and is generally caused by the compression of nerves or muscles. Limping, diminished range of motion, and other disabilities may be observed in the affected limbs.

### 1.2. Anatomical Pathology and Genetic and Molecular Patterns

Macroscopically, ASPSs are poorly delimited tumors with a soft consistency and a pale tan to yellow or gray–red–purple cut surface, often associated with hemorrhagic and necrotic areas [6]. Microscopically, tumor cells present a typical organoid, acinar, and alveolar arrangement. ASPS demonstrates a nested or organoid growth pattern, where the aggregates of tumor cells exhibit central degeneration, necrosis, and loss of cohesion, and the nests are divided by thin-walled vascular spaces [1,6]. Tumor cells are large, rounded, or more often polygonal, with eosinophilic granular to clear cytoplasm. The nucleus is vesicular, and the nucleolus is evident, with abundant granular, eosinophilic, and sometimes vacuolated cytoplasm. Mitotic figures are scarce [7]. The diagnostic hallmark is an unbalanced t(X;17)(p11;q25) translocation and its correspondent chimeric ASPSCR1-transcription factor E3 (TFE3) fusion protein [8,9]. Due to the infrequent retention of der(X) resulting from the t(X;17)(p11;q25), the der(17) t(X;17) could be characterized in certain instances as add(17)(q25). This is applicable when the quality of the banding is insufficient to positively identify the additional material as originating from the short arm of chromosome X [8]. This translocation leads to the fusion of the TFE3 transcription factor gene, located on Xp11, with ASPSCR1 (also known as ASPL) on 17q25.3. Depending on the specific TFE3 intron involved in the genomic rearrangement, ASPSCR1-TFE3 fusion transcripts may vary, either including or excluding an additional exon of TFE3 [9,10]. The ASPSCR1-TFE3 fusion protein is found in the nucleus, where it acts as an aberrant transcription factor that causes overexpression of c-Met and promotes c-Met signaling, making ASPS cells responsive to c-Met inhibition both in vitro and in vivo [8,9,10,11]. Schöffski et al. tested the c-Met inhibitor crizotinib in ASPS, and most patients experienced disease stabilization, although tumor size reduction was infrequent [12]. Another study in a mouse model demonstrated the oncogenic capacity of ASPSCR1-TFE3 fusion gene to induce sarcomagenesis in mice from conditional expression, highlighting a dependence of ASPS on lactate for growth [13]. This connection could be attributed to its frequent manifestation in muscular tissues and the elevated expression of the lactate transporter monocarboxylate transporter 1 (MCT1), alongside its associated protein, CD147 [13]. A recent study by Tanaka et al. has also demonstrated that ASPSCR1-TFE3 is also responsible for ASPS angiogenesis, inducing dynamic modifications in DNA super-enhancers related to the angiogenesis pathway, thus orchestrating ASPS vascular network construction [14]. Understanding these signaling pathways is fundamental in the management of these tumors, since ASPS traditionally shows resistance to conventional doxorubicin-based chemotherapy, so new treatment strategies have emerged in the form of monotherapy or combination therapy utilizing tyrosine kinase inhibitors and immune checkpoint inhibitors. These approaches have demonstrated antitumor activity and represent promising possibilities for treatment [15].

### 1.3. Imaging Features

Since ASPS tends to follow an indolent course, often leading to a presentation at an advanced stage, the identification of ASPS peculiar imaging features would be of great importance for a rapid referral to sarcoma centers for appropriate diagnosis, staging, and therapeutic management [4]. However, because of the rarity of ASPS, the radiological literature is limited and heterogeneous (different imaging tools analyzed inconsistently), mostly focusing on magnetic resonance imaging (MRI) and less frequently on computed tomography (CT) [5,16]. The few articles available in the literature regarding sonographic ASPS features describe the tumor appearance as a hypoechoic lobulated mass with well-defined margins and intra- and peri-lesional vessels, with high vascularization detected on color-Doppler evaluation. On CT imaging, ASPS shows low density on unenhanced scan and demonstrates intense contrast enhancement after contrast medium administration; CT also allows an optimal visualization of perilesional vessels. On MRI, ASPS demonstrates slight hyperintensity on T1 weighted imaging (WI) and hyperintensity on T2-WI, with peritumoral vessels and prominent vascularization. 

Therefore, in our series we aimed to analyze pre-treatment ASPS radiological characteristics on all available imaging modalities, comparing them with prior literature reports and metastatic patterns at diagnosis.

## 2. Materials and Methods

### 2.1. Study Design

This retrospective study included all patients with histologic diagnosis of ASPS performed by pathologists from our sarcoma reference center with at least one pre-treatment CT or MRI or ultrasonography (US) of diagnostic quality and brain–thorax–abdomen and pelvic CT for disease staging, treated at our cancer center between January 2006 and December 2021. Twelve patients affected by ASPS with local and baseline imaging studies were included in this study. This retrospective observational case series with a literature review was conducted in line with the guidelines of the local institutional review board. Informed consent was obtained from the subjects described in this report or from the next of kin (for minor patients) of subjects included in this report. The study was conducted according to the ethical standards set by the local ethics committees and according to the Declaration of Helsinki and subsequent amendments; the research was conducted in line with our local ethics committee guidelines. 

### 2.2. Clinical Data Collection

Clinical reports from our hospital were reviewed by a senior orthopedic oncology surgeon with 15 years of experience in the field (M.C.). The following data were collected from the medical files: patients’ age at diagnosis, sex, pain at diagnosis, tumor location, depth relative to the superficial fascia, and initial metastatic staging (baseline). Outcome data, when available, comprised the patient’s last status, categorized as non-evidence of disease (NED), alive with disease (AWD), died of disease (DOD), and dead unrelated to disease (DUD). Overall survival (OS) recorded in months was defined as the time between diagnosis and death related to illness or the patient’s last follow-up. 

### 2.3. Imaging Analyses

Two radiologists (P.S. and N.P.), with respectively 12 and 3 years of expertise in musculoskeletal oncologic imaging, reviewed in consensus all available radiological studies (images and reports) for local and distant staging on a Picture Archiving and Communications System (PACS-Carestream Vue PACS v. 11.4.1.1102, Philips healthcare, Amstelplein 2, 1096 BC Amsterdam, The Netherlands). The longest diameter (LD, considering the three planes) was measured on each modality. Signs of involvement of surrounding structures were recorded with regard to nerve/vessel encasement or bone erosion. The initially suspected diagnosis on baseline imaging studies reports were investigated as well.

#### 2.3.1. Ultrasonography Analysis

Ultrasound imaging studies were performed with Affiniti 50G (Philips, Amsterdam, The Netherlands; 2006–2020) or Logiq E10 (General Electric Healthcare, Chicago, IL, USA; 2020–2022).

We recorded the lesion echogenicity (categorized as homogeneous, focal inhomogeneous, or diffuse inhomogeneous), borders (categorized as well-defined, focally ill-defined, or ill-defined), presence of feeding vessels (present/absent), calcifications/mineralization (categorized as spindle-shaped, dot-shaped, or gross and amorphous), intravascularization on color-Doppler (categorized as mild, moderate, or strong), and Doppler sonographic pattern on peritumoral vessels (arterial signal or venous signal). 

#### 2.3.2. MRI Studies and Analysis

MRI imaging studies were performed with Signa HD, 1.5 Tesla (General Electric Healthcare, USA) using a standard protocol always including T1w sequence, T2w sequence, and one fluid sensitive sequence (T2w fat-saturated or Short tau inversion recovery—STIR).

Radiologists reported lesion MRI growth-pattern type (pushing, focal-infiltrative, or diffuse-infiltrative), signal intensity on T1-WI, T2-WI (defined as hyperintense, isointense, or hypointense compared to normal muscle), SI heterogeneity on T1-WI and T2-WI, signs of necrosis, fat, fibrosis, and hemorrhage. Surrounding tissues were evaluated in regard to peritumoral edema (absent, focal, diffuse), peritumoral enhancement (absent, present), and tail sign (absent, present). Peritumoral feeding vessels were studied (categorized as proximal, distal, or proximal and distal). The presence/absence of flow voids within peritumoral feeding vessels and within intratumoral vessels was recorded, as well as their number and dimensions (in millimeters). 

#### 2.3.3. CT Studies and Analysis

All CT scans were performed with contrast medium injection (standard iodinate contrast medium with a dose of 1.5–2.0 mL/kg), with a CT-Brilliance 16 slices (Philips Healthcare, The Netherlands) or with a Dual-Energy Revolution CT, 128 slices (General Electric Healthcare, USA). 

We analyzed tumor density on unenhanced scans (defined as hyperdense, isodense, or hypodense compared to normal muscle), homogeneity on unenhanced and contrast-enhanced scans (homogeneous, focal inhomogeneous, or diffuse inhomogeneous), the pattern of contrast enhancement (defined as mild, moderate, or strong), calcifications/mineralization (categorized as spindle-shaped, dot-shaped, or gross and amorphous), and peritumoral feeding vessels (proximal, distal, or proximal and distal).

### 2.4. Statistical Analysis

The Chi-square Test and Fisher’s Exact Test were used to assess associations among tumor dimensions, single imaging feature expressed, and metastatic risk at baseline controls.

The univariable odds ratio (OR) with 95% confidence interval (CI) was calculated for each imaging feature analyzed to assess the risk of distant metastasis at diagnosis. 

Data were considered statistically significant when *p* < 0.05. The analyses were performed using IBM^®^ SPSS^®^ 25.

### 2.5. Literature Review Search

We performed a literature review on the PubMed database, including all articles focused on ASPS imaging, to compare our experience with previously reported literature. Case series and original research were considered, while single case reports were excluded. The PubMed database was searched up to 1 June 2023, using the string: “alveolar soft part sarcoma” AND “imaging”. Additionally, relevant keywords were used in different combinations for free-hand search, and the bibliography of selected articles was reviewed. Only clinical studies reporting the imaging features of ASPS were included.

## 3. Results

### 3.1. Clinical Data

**Figure 1:**
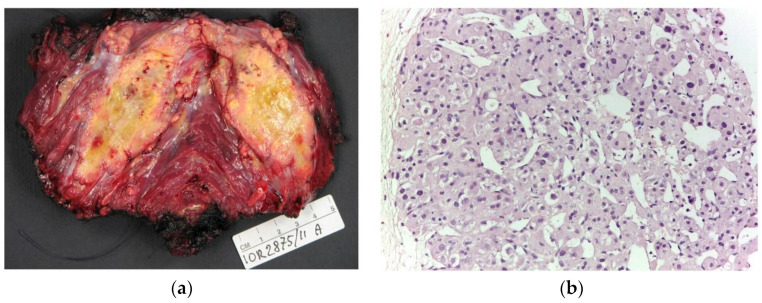
(**a**) Macroscopic appearance of alveolar soft part sarcoma, a poorly delimited soft tumor with yellowish cut surface. (**b**) Microscopically, ASPS shows a typical organoid, acinar, and alveolar arrangement of tumor cells with central degeneration (200× of magnification).

Twelve patients (7F, 5M; mean age 27.1, range 4–66 years old) with histopathological diagnosis of ASPS (Figure 1) and radiological studies available were included in the study.

**Figure 2:**
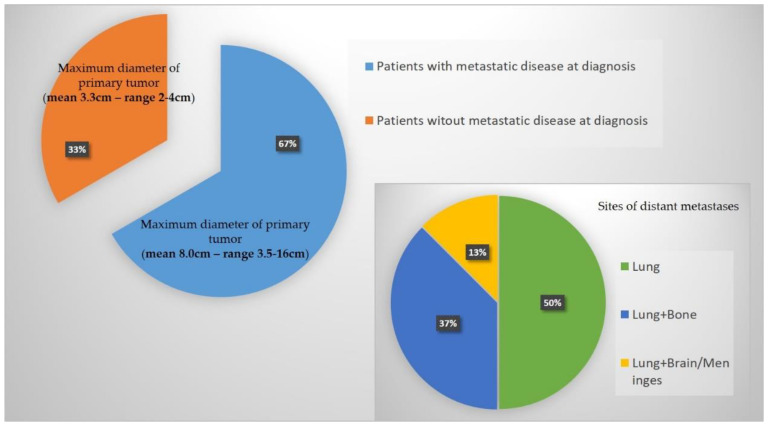
Graphical representation of main results in regards to distant metastases at diagnosis and maximum diameter of the primary tumor.

Both US and MRI were available in 7 patients (7/12—58.3%), MRI only in 3 patients (25%), and CT only in 2 patients (16.7%). Whole-body contrast-enhanced CT (head, thorax, and abdomen) was available in all patients (Table 1 and Figure 2). 

### 3.2. General Imaging Findings—Local and Distant Baseline Assessment

Eight out of twelve patients (66.7%) presented with distant metastases at diagnosis. In 4 of them (4/8—50%), the lungs were the only metastatic site at staging. Three patients (37.5%) had lung and bone metastases, while the remaining patient (12.5%) had lungs, brains, and meningeal lesions at diagnosis. All tumors were deeply seated (12/12). 

Pulmonary metastases appeared on CT and conventional radiography as well-defined, rounded solid nodules, often large and multiple. The pattern displayed is similar to the ones detectable on other STS-subtype lung metastases. On CT imaging, pulmonary metastases may show the “feeding-vessel” sign, indicating the appearance of the blood vessel supplying the nodule. No signs of calcification/mineralization were detected on pulmonary metastases.

The mean maximum diameter at baseline was 7.6 cm (range 2–10 cm). Patients without metastatic disease at diagnosis (4/12—33.3%) had a maximum diameter smaller than 5 cm (mean 3.3 cm—range 2–4 cm). In contrast, in patients with metastatic disease at diagnosis, the maximum diameter was greater than 5 cm (mean 8.0 cm— range 3.5–16 cm) in 7 patients (7/8—87.5%). A maximum diameter greater than 5 cm at diagnosis was significantly associated with the presence of distant metastases (*p* = 0.0101). A maximum diameter greater than 5 cm represents a risk of metastatic disease at diagnosis with an OR equal to 45.0000 (95% CI: 1.4908–1358.3585), *p* = 0.0285.

**Figure 3:**
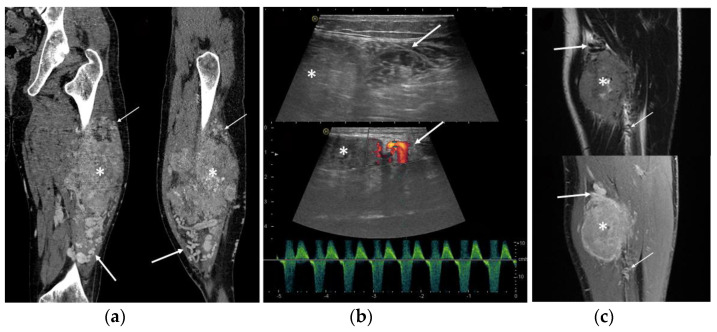
ASPS feeding vessels in different imaging modalities. Note the peritumoral tortuous vessels (arrows) proximally to the neoplasm (asterisk). (**a**) Coronal and sagittal view of CT scan; (**b**) ultrasound B-mode greyscale and color-Doppler with arterial pattern in the feeding vessels; (**c**) coronal views of T2-WI and contrast enhanced fat-saturated T1-WI.

Peritumoral tubular tortuous feeding vessels were found in all cases and identified by US (7/7 cases), MRI (10/10), and CT (3/3) (Figure 3). Peritumoral vessels were present proximally and distally to the neoplasm in 10/12 cases (83.3%) and at only one side of the lesion in the remaining two cases (Figure 3).

Calcifications/mineralization were detected in two patients (2/12—16.7%; one with US and one with CT), spot-like in one and gross-amorphous in the other. In only one patient (1/12—8.3%), bone erosion was detected (iliac wing involvement in a deep gluteal tumor). Radiological reports of baseline local assessment were doubtful in regard to the possible diagnosis in 11 patients (11/12—91.7%), while in one patient (8.3%), a suspected intramuscular hemangioma was suggested by radiologists on both US and MRI.

### 3.3. Ultrasound Features

**Figure 4:**
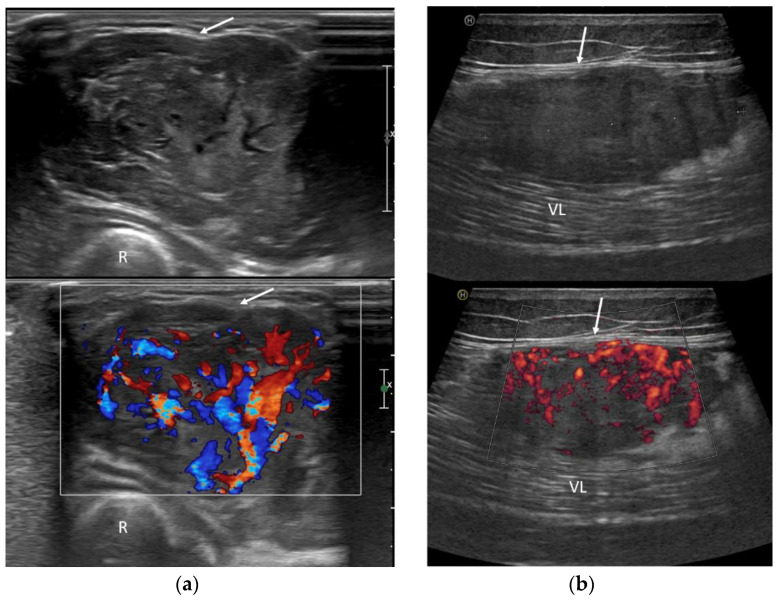
Ultrasound exams in two cases of ASPS demonstrate deep tumor localization in relation to the investing fascia (arrows) and rich neoplastic vascularization on color-Doppler assessment. (**a**) US images of the forearm (R: radius); (**b**) US images of the thigh (VL: vastus lateralis muscle).

US imaging revealed an inhomogeneous, prevalently hypoechoic pattern in all cases (7/7—100%). A rich neoplastic vascularization was also found in all patients, with abundant flow signals at color Doppler (Figure 4). The margins were always well-circumscribed (100%) and lobulated in two cases (2/7—28.6%). Peritumoral feeding vessels were detected in all cases (7/7—100%), and in five of them, Doppler sonography was recorded, always indicating an arterial pattern (5/5—100%) (Figure 4).

### 3.4. MRI Features

**Figure 5:**
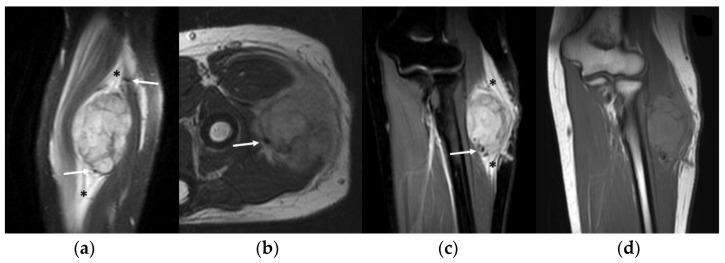
MRI of a 16-year-old female with alveolar soft part sarcoma of the forearm. Note the intra-/peritumoral flow voids on fluid-sensitive sequences can be noted as well (arrows). (**a**) Sagittal and (**c**) coronal Proton Density fat-saturated images demonstrate a moderately hyperintense neoplasm with diffuse peritumoral edema (asterisks); (**b**) axial T2-WI; (**d**) coronal T1-WI shows an inhomogeneous slightly hyperintense mass.

MRI studies revealed a mildly inhomogeneous slight hyperintense signal on T1-WI imaging and a moderately inhomogeneous hyperintense signal on T2-WI in all cases (10/10—100%). A ‘pushing type’ growth pattern was found in 7 cases (70%), while a focal (2 cases, 20%) or a diffuse (1 case, 10%) infiltrative type was found in the remaining MRI studies. A small central area of necrosis or a small central ‘stellar’ area of hyper-vascularization was detectable on contrast-enhanced sequences, respectively, of 4 patients (4/10—40%) and 2 patients (20%). Peritumoral edema on fluid-sensitive sequences was always present (10/10—100%), while peritumoral enhancement was detected in 2 patients (2/10—20%). Flow voids on fluid-sensitive sequences within the peritumoral feeding vessels, as well as within the mass, were found in all cases (10/10—100%) (Figure 5). The mean number of flow voids detected per single MRI study was 10 (range 6–15), while the mean dimension of flow voids was 3.5 mm (range 1–10 mm). The tail sign was detected in only 1 patient (1/10—10%)—Figure 5. 

### 3.5. CT Features

**Figure 6:**
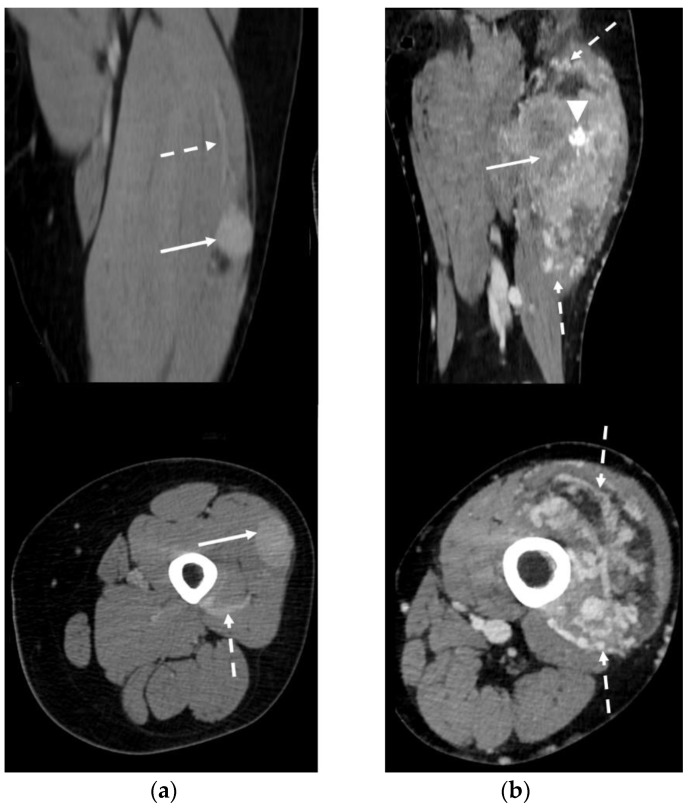
Contrast-enhanced CT in two patients affected by ASPS (arrows) of the thigh. Peri-/intratumoral feeding vessels can be clearly detected (dotted arrows). (**a**) Coronal and axial reconstructions demonstrate homogeneous lesion enhancement post contrast medium administration. (**b**) Coronal and axial reconstructions show a highly inhomogeneous enhancement pattern. Gross intratumoral calcifications can be noted (arrowhead).

In CT studies, ASPS presented as an inhomogeneous, slightly low-density mass on unenhanced scans, with a strong enhancement after intravenous contrast medium injection. The contrast-enhanced scan revealed a homogeneous pattern in 1 patient with a small lesion (3.5 cm—33.3%) and an inhomogeneous pattern in the remaining 2 patients (66.7%). The contrast-enhanced images detected peritumoral feeding vessels in all cases (3/3—100%). Intratumoral calcifications were identified in one of the three (33.3%) CTs available (Figure 6).

Imaging features constantly expressed in our series of 12 patients are summarized in Table 2.

### 3.6. Literature Review Results

Fourteen articles (case series or original research) focusing on ASPS imaging were found, with a total of 151 patients included [2,3,16,17,18,19,20,21,22,23,24,25,26,27,28]. We included studies with tumor location both at the extremities and in any other body region. 

A recent literature review performed by Li et al. reported that typical ASPS sonography features a heterogeneous hypoechoic appearance with well-defined margins, the presence of intra- and peri-tubular vessels, and high vascularization detected on color-Doppler assessment [26].

Regarding MRI studies, the slight hyperintensity on T1-WI and hyperintensity on T2-WI resulted in a main and consistently reported characteristic of ASPS. Intra- and peritumoral flow voids were reported in MRI results of 12 out of 14 studies (85.7%). Peritumoral vessels and high vascularization were always reported and recognized in different imaging modalities (contrast-enhanced MRI or CT, angiography).

Table 3 summarizes the main results from the studies (case series or original research) focused on ASPS imaging.

## 4. Discussion

ASPS is an ultra-rare subtype of STS of uncertain histogenesis, predominantly involving deep soft tissue of the extremities and mainly affecting the young population [30]. Chemotherapy is ineffective in the treatment of this neoplasm, while radiotherapy may play a role in reducing the risk of local recurrences after surgery [2]. Partial response to tyrosine kinase inhibitors, alone or in combination with checkpoint inhibitors or trabectedin, has been reported [31,32,33].

This study presents several limitations. First, the retrospective nature of the research and the small number of patients included due to the rarity of the disease impedes potential valuable analyses as a correlation between imaging features and patients’ prognosis. Additionally, the retrospective re-reading of the US examinations available can present some limitations due to the lack of real-time sonographic evaluation. A multivariate analysis of imaging-related prognostic factors was not feasible due to the small sample size.

We do not have any data regarding The Cancer Genome Atlas (TCGA) genomic mutations (in the current clinical scenario, the oncologist should request these). The diagnosis of each case described in this paper includes the molecular confirmation with the presence of t(X;17) ASPSCR1-TFE3 using RT-PCR and/or NGS (Fusion plex Archer sarcoma panel).

We presented the radiological features of ASPS patients treated at our sarcoma referral center over a period of 15 years. Our series, although limited in number, represents a significant radiological experience in the setting of an ultra-rare sarcoma, especially in regard to sonographic imaging features. All patients had at least CT or MRI at baseline, as per inclusion criteria.

In our series, the imaging features consistently found in the different imaging modalities were:

- Deep location
- Presence of peritumoral feeding vessels
- Inhomogeneous, mainly hypoechoic US pattern with strong internal vascularization at Color-Doppler evaluation
- Slight hyperintense MRI signal on T1-WI and a moderately inhomogeneous hyperintense signal on T2-WI
- MRI flow voids on fluid-sensitive sequences
- MRI peritumoral edema
- Slight low density and inhomogeneity on unenhanced CT

Combining these features with those already reported in other series, we can deduce that a slight hyperintense MRI SI on T1-WI, and hyperintense SI on T2-WI, numerous MRI flow voids, high vascularization (on contrast-enhanced MRI or CT, Doppler US, angiography), and peritumoral feeding vessels can be considered the hallmarks of ASPS imaging.

Given the malignant nature of ASPS, the identification of peculiar ASPS imaging features is consequently related to the possibility of achieving an early diagnosis with timely patient referral to sarcoma centers for prompt treatment [7]. As for other malignancies, the size of the tumor remains a key element that correlates with prognosis, even for this rare and aggressive subtype [34,35]. Indeed, in our series, a maximum diameter at diagnosis greater than 5 cm was always associated with metastatic disease at baseline staging. Radiologists should correctly suspect the malignant nature on baseline imaging to allow rapid and correct recognition of these tumors, suggesting histopathological diagnostic investigation. Particular attention should be paid to US features since US is frequently the first imaging modality performed in the assessment of soft-tissue lumps. In this regard, our results are consistent with those of Li et al. and concordant in defining the US appearance of ASPS as an inhomogeneous hypoechoic well-defined mass with strong vascularization [26]. Additionally, in our series, an arterial sonographic Doppler pattern was detected inside the peritumoral feeding vessels.

Peculiar ASPS imaging features should be well-known and considered in the differential diagnosis. In this regard, a recent multi-institutional research article demonstrated that some benign intramuscular hemangiomas and vascular malformations are the main differential diagnoses [16]. Indeed, even in our study, one patient had a radiological suspected diagnosis of intramuscular hemangioma on MRI and US evaluation. A slightly high signal intensity on MRI T1-WI, the presence of numerous flow voids (>5), and a deep location may suggest the suspicion of ASPS [8]. In US studies, the well-circumscribed margins displayed by ASPS can also help in the differential diagnosis of hemangiomas, which usually present with ill-defined borders. Nonetheless, a deep location was considered to be an element of possible misdiagnosis [16,36].

It is worth remembering that, for all soft-tissue masses with a deep-seated location, the absence of characteristic imaging features of the most common and recognizable benign conditions (e.g., ganglion cyst, simple lipoma, ossificans myositis) should lead to adequate assessment in specialized centers [37].

## 5. Conclusions

Our results are in line with the previous series focused on ASPS imaging features. The knowledge of these radiological presentations among radiologists and their distinction from benign conditions can provide an early diagnosis with rapid referral to a sarcoma center. A prompt diagnosis reflects a direct impact on a patient’s prognosis and metastatic risk. Future multi-institutional research is recommended in order to better define ASPS imaging features as well as to describe potential imaging features related to prognosis.

## Figures and Tables

**Table 1:** Patients’ main characteristics, imaging studies available, tumor sizes, and metastatic patterns. Patients’ current status: non-evidence of disease (NED), alive with disease (AWD), died of disease (DOD).

Patient n°	Age, Sex	Symptoms	Lesion Location (Deep/Superficial)	Baseline Imaging Tools Available	Longest Diameter (cm)	Metastasis at Diagnosis (Sites)	Current Status	Survival (Months)
1	24, M	Non-painful lump	Leg (deep)	US, MRI	6	Yes (lung)	AWD	46
2	20, F	Non-painful lump	Popliteal fossa (deep)	MRI	3.5	No	NED	144
3	16, F	Painful lump	Forearm (deep)	US, MRI	4	No	NED	41
4	44, M	Painful lump	Thigh (deep)	MRI	7	Yes (lung, bone)	DOD	135
5	66, F	Painful lump	Hip girdle (deep)	MRI, CT	8	Yes (lung, bone)	DOD	24
6	26, F	Painful lump	Hip girdle (deep)	CT	10	Yes (lung, brain, meninges)	DOD	39
7	20, M	Non-painful lump	Thigh (deep)	US, MRI	8	Yes (lung, bone)	DOD	6
8	19, F	Non-painful lump	Leg (deep)	US, MRI	3.5	Yes (lung)	AWD	6
9	25, F	Non-painful lump	Thigh (deep)	US, MRI, CT	3.5	No	NED	32
10	35, M	Painful lump	Thigh (deep)	CT	16	Yes (lung)	DOD	1
11	4, F	Painful lump	Arm (deep)	US, MRI	2	No	NED	9
12	23, M	Non-painful lump	Thigh (deep)	US, MRI	5.5	Yes (Lung)	DOD	165

**Table 2:** Summary of the imaging features constantly displayed in our series on multimodality imaging. CE: contrast enhanced; SI: signal intensity.

General Imaging Features	US	CE MRI	CE CT
Deep-seated location	Well-defined borders	Intra and peritumoral flow-voids	Low density on unenhanced scans
Peritumoral feeding vessels	Inhomogeneous hypoechoic pattern	Slightly high SI on T1w	-
Strong internal vascularization	Arteriosus Doppler sonographic pattern inside peritumoral vessels	High SI on T2w	-

**Table 3:** ASPS imaging reported in 14 previous retrospective studies evaluated in our literature review. SI = signal intensity; CE = contrast enhancement; NA: not available.

First Author, Year, Reference Number	Number of Patients	Age (Years)	Average Longest Diameter	Metastatic Disease at Diagnosis	Baseline Imaging	Main Imaging Findings
Iwamoto, 1995, [25]	10	11–40	NA	2/10 (20%)	MRI, Angiography	MRI: Slightly high SI on T1w, high SI on T2w. Flow voids.
Kim, 2005, [29]	5	4–22	72 mm	7/10 (70%)	CT, MRI, Angiography	CT: Strong CE. MRI: Slightly high SI on T1w, high SI on T2w. Flow voids. Angiography: Hypervascular lesion.
Park, 2010, [21]	3	NA	NA	1/5 (20%)	MRI	MRI: Slightly high SI on T1w, high SI on T2w. Flow voids.
Viry, 2013, [18]	6	7–17	NA	3/3 (100%)	CT, MRI	CT: Low density on unenhanced scans. MRI: Slightly high SI on T1w, high SI on T2w. Highly vascularized lesions. Intra-/peritumoral vessels with high flow (=flow voids). Central stellar necrotic component or central stellar highly vascular area (for >7 cm tumors).
Li, 2014, [16]	14	27–54	98 mm	4/5 (80%)	MRI	MRI: Slightly high SI on T1w, high SI on T2w. Flow voids. Inhomogeneous signal intensity.
Suh, 2014, [23]	10	17–48	NA	8/14 (57.1%)	MRI, Angiography	MRI: Slightly high SI on T1w, high SI on T2w. Flow voids. Inhomogeneous SI. Angiography: Peritumoral vessels with arteriovenous shunts.
McCarville, 2014, [22]	22	8–23	59 mm	11/22 (50%)	CT, MRI	MRI: Slightly high SI on T1w, high SI on T2w. Flow voids. Nodular internal architecture, separated by thin hypodense bands. Intense or moderate CE with central necrosis. CT: Intra- and peritumoral vessels. Highly vascularized lesions on CE scan.
Qiao, 2015, [19]	6	16–45	48 mm	Not reported	CT, MRI	CT: Highly vascularized on CE scan. Hypodense lesions on unenhanced scan. MRI: Hypointense on T1w, hyperintense on T2w.
Tian, 2016, [17]	14	13–37	91 mm	Not reported	CT, MRI	CT: Low density on unenhanced scan. MRI: Slightly high SI on T1w, high SI on T2w. Highly vascularized lesions. Intra-/peritumoral vessels. Flow voids.
Sood, 2016, [28]	25	18–40	102 mm	18/25 /72%)	CT, MRI	CT: Low density on unenhanced scan. MRI: Slightly high SI on T1w, high SI on T2w. Highly vascularized lesions. Intra-/peritumoral vessels. Flow voids.
Cui, 2017, [20]	12	21–34	68 mm	5/12 (41.7%)	MRI	MRI: High signal intensity on T2w. Highly vascularized lesions. Flow voids. Central area of necrosis or hypervascularization.
Crombé, 2018, [16]	25	7–53	66 mm	14/25 (56%)	MRI	MRI: Slightly high SI on T1w, high SI on T2w. Infiltrative growth pattern. Deep location. Tubular feeding vessels. Flow voids (>5). Central area of necrosis. Absence of tail sign, absence of fibrotic signal.
Li, 2022, [24]	3	23–30	81 mm	2/3 (66.6%)	US, MRI, CT	US: Heterogeneous hypoechoic tissue. Well-defined margins. Intra- and peri-tubular vessels. Highly vascularized lesions on color-Doppler. MRI: Slightly high SI on T1w, high SI on T2w. Flow voids. CT: Slightly high density without significant bony destruction on CT.
Gulati, 2021, [27]	16	3–72	83 mm	14/16 (87.5%)	US, CT, MRI, PET	PET: SUV of >2.5. CT: Intense CE. MRI: Slightly high SI on T1w, high SI on T2w. Flow voids. Intense CE. US: Circumscribed lobulated homogeneously hypoechoic pattern. Multiple enlarged feeding vessels.

## Data Availability

The data presented in this study are available on request from the corresponding author.
